# Volatilomic Signatures of Parental and Oxaliplatin-Resistant HCT116 Colon Cancer Cell Lines

**DOI:** 10.3390/ijms27146240

**Published:** 2026-07-13

**Authors:** Christine Heinzle, Andreas Leiherer, Axel Muendlein, Clemens Ager, Agnieszka Królicka, Chris A. Mayhew, Pawel Mochalski

**Affiliations:** 1 Vorarlberg Institute for Vascular Investigation and Treatment (VIVIT), 6800 Feldkirch, Austria; andreas.leiherer@vivit.at (A.L.); axel.muendlein@vivit.at (A.M.); 2 Central Medical Laboratories, 6800 Feldkirch, Austria; 3 Faculty of Medical Sciences, Private University in the Principality of Liechtenstein, FL-9495 Triesen, Liechtenstein; 4 Institute for Breath Research, University of Innsbruck, 6020 Innsbruck, Austria; clemens.ager@uibk.ac.at (C.A.); christopher.mayhew@uibk.ac.at (C.A.M.); 5 Department of Building Materials Technology, Faculty of Materials Science and Ceramics, AGH University of Science and Technology, 30-059 Krakow, Poland; krolicka@agh.edu.pl; 6 Institute of Chemistry, Jan Kochanowski University, 25-369 Kielce, Poland

**Keywords:** colorectal cancer, cancer metabolism, volatilomics, volatile organic compounds, HS-NTE-GC-MS, oxaliplatin resistance

## Abstract

Volatile organic compounds (VOCs) reflect cellular metabolic activities and may serve as non-invasive biomarkers in oncology. This study investigated whether acquired oxaliplatin resistance in colorectal cancer is associated with distinct volatilomic alterations. The human colorectal cancer cell line HCT116 and its oxaliplatin-resistant derivative (OXrHCT116) were analyzed under basal conditions and following oxaliplatin exposure. Chemoresistance was confirmed using dose–response, cell viability, and colony formation assays. VOCs were analyzed by headspace needle trap extraction coupled with gas chromatography–mass spectrometry (HS-NTE-GC-MS). OXrHCT116 cells exhibited markedly reduced oxaliplatin sensitivity, increased IC_50_ values, and reduced proliferative and clonogenic capacity. Volatilomic profiling identified 55 significantly altered VOCs. Parental HCT116 cells displayed broader VOC diversity and higher turnover than OXrHCT116 cells. Hydrocarbons associated with lipid peroxidation and oxidative stress were more abundant in HCT116 cells, whereas resistant cells showed markedly reduced emission of these compounds. Additional alterations in aldehydes, alcohols, and aromatic compounds suggested reduced metabolic flux in resistant cells. Oxaliplatin exposure induced pronounced volatilomic changes in HCT116 cells but only minimal modulation in OXrHCT116 cells. These findings suggest that oxaliplatin resistance may be associated with distinct metabolic reprogramming and support VOC profiling as a promising approach for monitoring chemoresistance.

## 1. Introduction

Volatile organic compounds (VOCs), collectively referred to as a “volatilome”, play an important role in the human organism and reflect a wide range of physiological and pathological processes. Human VOCs have attracted increasing attention due to their potential to provide information about the presence of disease or infection, thereby offering opportunities for non-invasive diagnostic applications. This potential arises because pathological processes in the human body alter the volatile signatures through mechanisms such as upregulation or downregulation of enzyme activity, oxidative stress, gene activation or repression, and changes in the microbiota. Consequently, VOCs can serve as indicators for early signs of disease, including colorectal cancer (CRC) [1,2,3].

Volatilomics aims to capture these disease-associated alterations by analyzing the volatiles emitted from various bodily excretions, including breath, skin, urine, saliva, and sweat. The non-invasive nature of VOC collection constitutes a major advantage compared to invasive clinical sample collection and underpins the development of novel, easy-to-use, and efficient diagnostic tools for clinical practice. Importantly, VOC signatures can be assessed using portable, low-cost, and user-friendly analytical platforms, such as miniature chemical sensor-based devices.

As a diagnostic tool, VOC analysis can function as an initial screening or triage method, potentially reducing reliance on more invasive and costly clinical diagnostic procedures. For these reasons, volatilomic profiling has been the subject of intense research in recent years [4,5].

CRC is among the most common malignancies worldwide and remains a leading cause of cancer-related mortality despite significant advances in screening and treatment strategies [6]. In advanced CRC, standard first-line chemotherapy frequently includes oxaliplatin-based regimens, such as FOLFOX (combination of folinic acid, fluorouracil, and oxaliplatin), which have substantially improved patient outcomes [7,8]. However, the long-term clinical efficacy of oxaliplatin is often limited by the development of acquired chemoresistance, which represents a major challenge in CRC management [9].

Oxaliplatin exerts its cytotoxic effects primarily through the formation of DNA adducts, leading to DNA damage and the induction of apoptosis [10,11]. Cancer cells can evade these effects through multiple resistance mechanisms, including enhanced DNA repair capacity, altered drug uptake and efflux, and the activation of detoxification pathways [9]. Increasing evidence further suggests that metabolic reprogramming and redox adaptation play a crucial role in the development of chemoresistance, enabling tumor cells to survive under cytotoxic stress conditions [12].

Understanding metabolic alterations associated with oxaliplatin resistance may facilitate the identification of novel biomarkers and therapeutic targets. In this context, the analysis of VOCs, which directly reflect cellular metabolic activity, has emerged as a promising approach to characterize tumor-specific metabolic phenotypes and treatment responses [13,14].

In this study, we focused on the volatilomic profiles of parental and oxaliplatin-resistant HCT116 CRC cell lines to identify resistance-associated differences in their volatilomes. HCT116 cells are a well-established CRC model frequently used in studies of oxaliplatin sensitivity and resistance, making them a suitable system for the present work. Specifically, we aimed to characterize VOCs produced or consumed by these cells and to identify metabolic alterations associated with oxaliplatin resistance. Such volatilomic signatures may improve our understanding of chemoresistance-associated metabolic pathways. Moreover, both cell lines were exposed to oxaliplatin to identify volatilomic changes induced by this drug. These findings may facilitate the identification of VOC markers of oxaliplatin resistance and support future non-invasive monitoring approaches in CRC.

To achieve these goals, headspace needle trap extraction (HS-NTE), for pre-concentration and gas chromatography with mass spectrometric detection (GC-MS) were employed to respectively capture and analyze the headspace (HS) above the cell culture system.

## 2. Results

### 2.1. Dose–Response of Oxaliplatin

**Figure 1:**
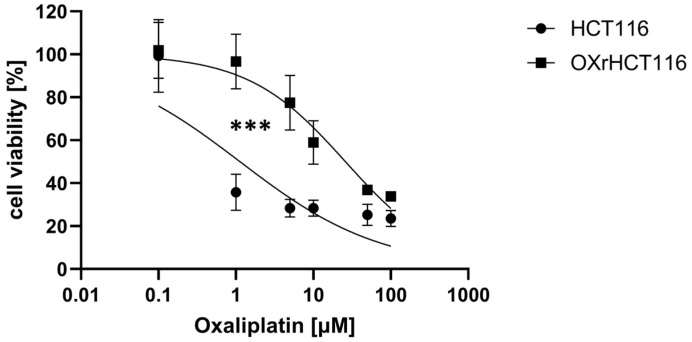
Dose–response curve of oxaliplatin in HCT116 and OXrHCT116 cells. Data represents the mean ± SD of 3 independent replicates. Dose–response curves were fitted by nonlinear regression using a variable-slope model. Statistical differences between the curves were evaluated using an extra sum-of-squares F test (*** *p* < 0.001).

Dose–response analysis revealed a pronounced difference in oxaliplatin sensitivity between HCT116 and OXrHCT116 cells (Figure 1). Parental HCT116 cells exhibited a marked reduction in viability at low oxaliplatin concentrations, whereas OXrHCT116 cells displayed a clear rightward shift of the dose–response curve, indicative of reduced drug sensitivity. Nonlinear regression analysis confirmed that the two curves were highly significantly different (extra sum-of-squares, F test, *p* < 0.001; F (2,29) = 27.93). The calculated IC_50_ value was substantially higher in OXrHCT116 cells (25.98 µM, 95% CI: 17.07–41.21 µM) compared to parental HCT116 cells (0.54 µM, 95% CI: 0.42–2.51 µM), representing an approximately 48-fold increase in oxaliplatin resistance.

### 2.2. Cell Viability of HCT116 and OXrHCT116

**Figure 2:**
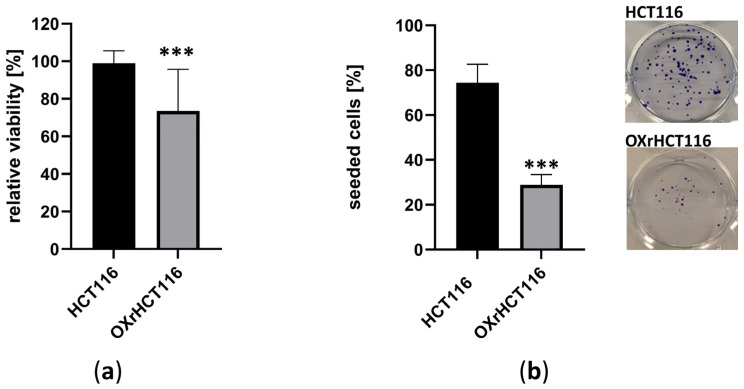
Proliferative and clonogenic capacity of HCT116 and OXrHCT116 cells. Data are presented as mean ± SD of 3 independent replicates. *** denotes statistically significant differences compared with parental HCT116 cells (*p* < 0.001). (**a**) MTT assay showing relative cell viability (%) of HCT116 and OXrHCT116 cells. Values were normalized to the mean viability of parental HCT116 cells, which was set to 100%. (**b**) Colony formation assay showing colony outgrowth, expressed as the percentage of seeded cells. Representative images of stained colonies are shown for both cell lines.

To assess differences in proliferative capacity between parental HCT116 and oxaliplatin-resistant OXrHCT116 cells, MTT and colony formation assays were performed in the absence of oxaliplatin (Figure 2). Both assays revealed a highly significant reduction in cell growth in OXrHCT116 cells compared with HCT116 cells (*p* < 0.001) corresponding to a very large effect size for cell viability (Cohen’s d = 1.55) and an exceptionally large effect size for clonogenic growth (Cohen’s d = 6.78). Consistent with these findings, colony formation assays demonstrated a markedly reduced clonogenic potential in OXrHCT116 cells, characterized by both a decreased number and smaller size of colonies. Representative images illustrate the diminished colony-forming ability of the oxaliplatin-resistant cell line.

Collectively, despite their increased oxaliplatin resistance, OXrHCT116 cells exhibit reduced proliferative and clonogenic capacity under standard culture conditions. To account for these growth differences, a higher number of OXrHCT116 cells was seeded for subsequent VOC analyses to ensure comparable overall cell numbers at the time of measurement.

### 2.3. Volatilome Signatures of Cell Lines Under Study

**Figure 3:**
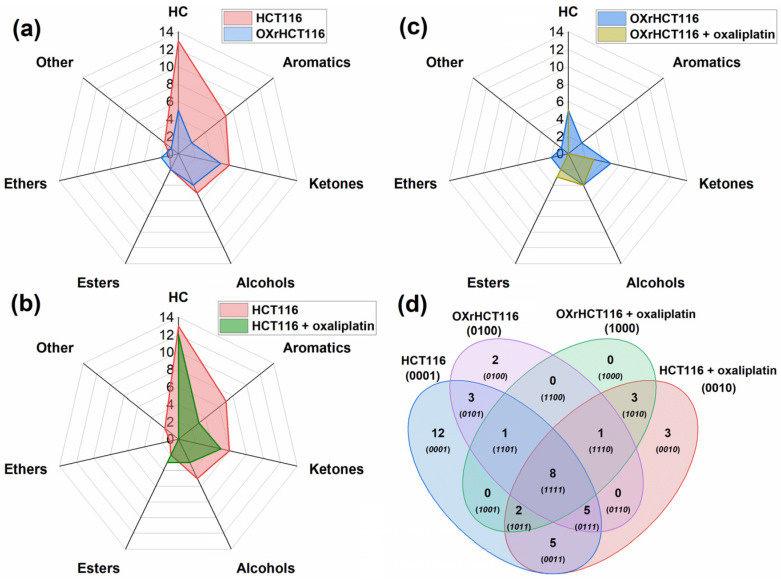
Volatilomic profiles of HCT116 and OXrHCT116 cells. (**a**) Distribution of VOCs emitted by parental and oxaliplatin-resistant HCT116 cells according to chemical class. (**b**) Distribution of VOCs emitted by parental HCT116 cells under control conditions and after oxaliplatin exposure, grouped by chemical class. (**c**) Distribution of VOCs emitted by OXrHCT116 cells under control conditions and after oxaliplatin exposure, grouped by chemical class. (**d**) Venn diagram showing the cumulative number of unique VOCs emitted by parental and oxaliplatin-resistant HCT116 cells under basal conditions and after oxaliplatin exposure. Numbers in parentheses denote the binary code identifying the experimental groups represented in each intersection (0001 = HCT116, 0010 = HCT116 + oxaliplatin, 0100 = OXrHCT116, 1000 = OXrHCT116 + oxaliplatin).

Among the detected volatiles, 55 showed statistically significant differences in their HS levels in at least one cell line compared to medium-only controls. Of these, 11 compounds exhibited decreased HS concentrations, whereas the remaining 44 showed elevated concentrations. The detection incidences of the identified volatiles and the concentrations of the VOCs in the HS of the cultivation flasks are summarized in Table S1 (Supplementary Materials). The results of the Wilcoxon signed-rank test are presented in Table 1 and Table 2, whereas the relative distributions of released VOCs according to their chemical class are shown in Figure 3. It should be noted that the chemical identities of some VOCs could not be fully confirmed by retention time matching and therefore remain putatively identified.

The 11 VOCs found to have decreased HS levels were 2 aldehydes (2-methylbutanal, 3-methylbutanal), 5 hydrocarbons (3,4-dimethyl-1-octene, 4,5-dimethyl-1-hexene, 2,3,5,8-tetramethyl-decane, 3,4-dimethyl-1-decene, and 5-tridecene), 3 alcohols (2-butyl-1-octanol, 4-ethyl-1-octyn-3-ol, 2,4-hexadien-1-ol), and 1,3-dioxan-5-ol.

The consumption of 2-methylbutanal and 3-methylbutanal is frequently observed in cultures of human cell lines both parental and mutated [15]. Two metabolic routes may be involved in their bioconversion: (i) oxidation into the corresponding carboxylic acids, mediated by enzymes with overlapping substrate specificities, such as aldehyde dehydrogenases (ALDHs), aldehyde oxidases (AOXs) and xanthine oxidoreductases (XOs); and (ii) reversible reduction into alcohols catalyzed by alcohol dehydrogenases (ADHs) [16,17,18]. Consistent with this, the potential reduction product of 3-methylbutanal, 3-methyl-1-butanol, was detected in the HS of both culture medium and cell cultures. However, its production by the investigated cells could not be statistically confirmed.

Analogously, the uptake of the aforementioned alcohols may be attributed to the synergy of ADHs/cytochrome P450 and ALDHs/AOXs/XOs.

Parental HCT116 cells metabolized several unsaturated hydrocarbons. The metabolism of VOCs belonging to this chemical class can be ascribed to the activity of cytochrome P450 isoforms, such as CYP2E1, which have been shown to oxidize a wide range of volatiles, including unsaturated hydrocarbons [19,20].

VOCs identified as being produced by cellular metabolism included 18 hydrocarbons (16 alkanes), 7 ketones, 5 alcohols, 7 aromatic compounds, 3 esters, 2 ethers, and 2 chlorine-containing compounds.

The metabolic pathways leading to alkane formation in humans are unclear; however, it can be assumed that these compounds arise from lipid peroxidation of polyunsaturated fatty acids (PUFA) induced by oxidative stress [13]. There is substantial evidence that lipid peroxidation of omega-3 and omega-6 fatty acids results in the production of ethane and n-pentane [21,22]. More specifically, these alkanes are generated via β-scission of alkoxy radicals formed during the homolytic cleavage of fatty acids hydroperoxides. For example, n-pentane has been shown to originate from the peroxidation of linoleic and arachidonic acids [22]. Other alkanes have been postulated to be produced through analogous mechanisms [23,24]. Interestingly, the release of saturated hydrocarbons has been reported in gastric mucosa cell lines, including both non-malignant (GES-1) and cancerous (AGS, SNU-1) cell lines [15].

Seven aromatic compounds were observed to be liberated by parental HCT116 cells, including benzene, toluene, o-xylene, ethylbenzene, styrene, 1,3-dimethylbenzene, and 1,2,3-trimethylbenzene. In contrast, oxaliplatin-resistant HCT116 cells emitted only two VOCs from this chemical class, namely styrene and 1,3-dimethylbenzene. The metabolic origin of these aromatic VOCs remains unclear.

Seven ketones, predominantly methyl ketones (five compounds), were identified as products of cellular metabolism, including acetone, 2-butanone, 2-pentanone, 3-pentanone, 2-heptanone, 4-methyl-2-pentanone, and cyclohexanone. The production of ketones in humans can be attributed to the oxidation of secondary or cyclic alcohols mediated by ADHs and/or cytochrome P450 [17]. For example, 2-butanone may arise from the oxidation of 2-butanol, while 2-pentanone may originate from 2-pentanol. However, among the putative alcohols, only 2-butanol and cyclohexanol were detected in the cultures under study. It is possible that other precursor alcohols had been consumed by cells under study at the time of measurement, thereby resulting in concentrations below the detection limits. These alcohol intermediates could also be produced by the cells via hydroxylation of corresponding alkanes catalyzed by cytochrome P450 enzymes [20,25,26]. This hypothesis is supported by the wide range of potential alkane substrates detected in both the culture medium and the cellular HS. For instance, n-heptane and cyclohexane were detected in the HS, whereas the cells were shown to produce n-pentane. Thus, n-pentane could be metabolized into 2-pentanol (84%), or 3-pentanol (15%) [20]. In addition, ketones in humans have also been reported to arise during the β-oxidation of fatty acids. For example, 2-ethylhexanoic acid is metabolized into 2-heptanone and 4-heptanone [27], and 2-pentanone is postulated to be formed from hexanoic acid via a peroxisomal pathway [26]. Furthermore, 3-pentanone could be a product of the oxidative decarboxylation of 2-methyl-3-ketovaleric acid [28]. It is plausible that other ketones observed in this study were produced through similar metabolic pathways.

With respect to alcohols, five compounds belonging to this chemical class—1-propanol, 2-methyl-1-propanol, 2-methyl-2-propanol, 2-methyl-2-butanol and 2-ethyl-1-hexanol—were found to be produced by parental and oxaliplatin-resistant HCT116 cells. These alcohols may arise from the reversible reduction of aldehydes or ketones driven by ADHs. Thus, 2-methyl-1-propanol could be a reduction product of 2-methyl-propanal, whereas 1-propanol may be derived from propanal. Secondary alcohols such as 2-methyl-2-propanol can be produced via two main pathways: (i) hydroxylation of corresponding branched alkanes [20,29] and (ii) biotransformation of ethers [30]. The hydroxylation of branched alkanes is mediated by cytochrome P450 isoforms CYP1A2, CYP2B6, and CYP2E1, and occurs predominantly at secondary or tertiary C-H bonds [20]. In line with this pathway, 2-methylpropane is converted into 2-methyl-2-propanol, while 2-methylbutane is hydroxylated predominantly into 2-methyl-2-butanol (approximately 70%) and, to a lesser extent, 3-methyl-2-butanol (approximately 25%). Unfortunately, 2-methylpropane is too volatile to be detected using the applied analytical method, and it is therefore unclear whether this pathway contributed to the observed release of 2-methyl-2-propanol. In contrast, 2-methylbutane was detected in the HS of both the cell cultures and culture medium. A similar hydroxylation pathway may also account for the formation of cyclohexanol and its subsequent oxidation to cyclohexanone [29]. An alternative mechanism for secondary alcohol formation involves the cytochrome P450-mediated biotransformation of aliphatic ethers [30]. In this pathway, 2-ethoxy-2-methylpropane and 2-methoxy-2-methylpropane yield 2-methyl-2-propanol, whereas 2-methoxy-2-methylbutane is metabolized into 2-methyl-2-butanol. Notably, 2-ethoxy-2-methylpropane was detected as a product of both parental and oxaliplatin-resistant HCT116 cells. The production of 2-ethyl-1-hexanol can stem from three pathways: (i) the metabolism of di(2-ethylhexyl) phthalate (DEHP), (ii) oxidation of 2-ethyl-hexanal catalyzed by ADHs, or (iii) enzymatic hydrolysis of 2-ethylhexyl benzoate [31]. DEHP, a plasticizer used in polyvinyl chloride, is hydrolyzed in humans to mono(2-ethylhexyl) phthalate (MEHP) and 2-ethylhexanol by cholesterol esterase (CEase), and/or carboxylesterase Ces1e [32]. Subsequently, 2-ethylhexanol is next oxidized to 2-ethylhexanoic acid and further metabolized to 2-heptanone and 4-heptanone [27,33]. However, as the presence of DEHP in the applied culture medium could not be verified and neither 2-ethylhexyl benzoate nor 2-ethyl-hexanal was detected in the HS of the cell cultures, the origin of 2-ethylhexanol remains unresolved.

Three ethyl esters—ethyl acetate, ethyl propanoate and ethyl 2-methylbutyrate—were identified as products of cellular metabolism. Notably, ethyl 2-methylbutyrate was released only in the presence of oxaliplatin. These esters may arise from esterification reactions involving ethanol present in the culture medium and the corresponding carboxylic acids, namely acetic acid, propanoic acid and 2-methylbutanoic acid. The latter acids could be generated through the oxidation of respective aldehydes and/or alcohols mediated by ADHs, cytochrome P450 enzymes (e.g., CYP2E1), and ALDHs/AOXs/XOs. For instance, acetic acid is a well-established product of ethanol oxidation. Nevertheless, alternative biochemical pathways, such as the Krebs cycle or pyruvate metabolism, cannot be excluded. Similarly, propanoic acid and 2-methylbutanoic acid are presumably products of the oxidation of propanal and 2-methylbutanal, respectively.

The metabolic routes leading to the production of the two ethers 2-ethoxy-2-methylpropane and 1,1-diethoxy ethane released by the cells under study are unknown.

### 2.4. Comparison of Volatilomic Signatures of Parental and Oxaliplatin-Resistant HCT116 Lines

Within the limitations of this study, several meaningful insights can nevertheless be extracted regarding metabolic differences between the investigated cell lines. Notably, only eight compounds were emitted by all four experimental groups. Furthermore, parental HCT116 cells released substantially more VOCs than the oxaliplatin-resistant OXrHCT116 cells (36 vs. 21 compounds, respectively). A similar trend was observed following oxaliplatin exposure, with 27 VOCs detected in HCT116 cultures compared with 15 in OXrHCT116 cultures. A similar pattern was evident for compounds uptake: parental HCT116 cells were found to consume 8 VOCs, whereas the oxaliplatin-resistant cells metabolized only 2. Due to the limited availability of detailed information on the enzyme activity and expression in HCT116 cells, definitive mechanistic conclusions cannot be drawn. However, these findings may be consistent with a lower overall metabolic activity in OXrHCT116 cells.

Interestingly, the observed volatilome profiles of parental and oxaliplatin-resistant HCT-116 cells exhibited significant divergence. Parental HCT116 cells emitted 12 unique VOCs, as shown in Figure 3.

Moreover, compared to OXrHCT116 cells, parental HCT116 cells displayed a markedly higher emission of (i) hydrocarbons (13 vs. 5 compounds), (ii) aromatic compounds (7 vs. 2 compounds) and (iii) a greater capacity to consume unsaturated hydrocarbons.

It is noteworthy, that of the 13 hydrocarbons released by parental HCT116 cells, all but one were alkanes, mainly branched species (9 compounds). In contrast, the oxaliplatin-resistant HCT116 line emitted only 5 alkanes. Although the metabolic pathways leading to alkane production in humans are unclear, they are postulated to be produced during the peroxidation of PUFA mediated by oxidative stress. Accordingly, the observed differences in alkane emission may reflect variations in the level of oxidative stress between parental and oxaliplatin-resistant HCT116 cells. It should further be emphasized that breath alkanes and methylated alkanes have been previously proposed as breath biomarkers of oxidative stress in lung cancer [24], blood cancer [34], and breast cancer [35].

As noted above, the metabolic origin of aromatic compounds emitted by the investigated cell lines is unclear. However, recently β-glucuronidase, an enzyme prevalent in the tumor microenvironment, was demonstrated to metabolize phenyl-β-D-glucuronide into phenol [36]. Perhaps other aromatic VOC could be produced in an analogous way in cancer cells. Nevertheless, the release of aromatic VOCs by human cells has been reported in several other studies. For example, benzene and styrene have been reported to be liberated by A549 lung cancer cells [37,38], as well as by AGS and SNU-1 gastric cancer cells [31]. Toluene was found to be emitted by human endothelial cells (HUVEC) [39]. Moreover, benzene release has also been observed in the gastric cancer cell line HGC-27 [40], and in AGS and SNU-1 cells [31].

It is further noteworthy that, contrary to oxaliplatin-resistant HCT116 cells, parental HCT116 cells metabolized several unsaturated hydrocarbons. This difference may reflect variations in the expression or activity of cytochrome P450 isoforms between the two cell lines.

Interestingly, in the presence of oxaliplatin, parental HCT116 cells exhibited a reduced emission of aromatic compounds. Given that the metabolic origin of the aromatic VOCs observed in this study is unclear, interpretation of this finding is challenging. In contrast, exposure of OXrHCT116 cells to oxaliplatin did not markedly alter their volatilome profile, as shown in Figure 3c. The only pronounced response of the oxaliplatin-resistant cells to chemotherapeutic stress was a reduction in the emission of methyl ketones (2 vs. 5).

## 3. Discussion

The present study demonstrates that acquisition of oxaliplatin resistance in HCT116 cells is associated with pronounced alterations in both cellular behavior and volatilomic profiles. Specifically, OXrHCT116 cells exhibited a significantly reduced proliferative and clonogenic capacity, as shown by MTT and colony formation assays, alongside a marked shift in VOC emission patterns.

A key finding of this study is the substantially lower number of both emitted and consumed VOCs in OXrHCT116 cells compared to parental HCT116 cells. This observation may reflect reduced metabolic activity in the oxaliplatin-resistant cell line, which is consistent with its decreased proliferation rate. Although seeding densities were adjusted to obtain comparable cell numbers at the time of VOC sampling, differences in growth kinetics and metabolic activity may still have influenced VOC production. Previous studies have demonstrated that VOC emissions are closely linked to cellular metabolism and cell expansion, suggesting that proliferation-dependent metabolic changes may influence VOC production independent of chemoresistance [41,42]. Consequently, some of the observed VOC alterations may reflect differences in cellular growth state rather than resistance-associated mechanisms alone. Nevertheless, reduced proliferative capacity of oxaliplatin-resistant colorectal cancer cells has been reported previously [43,44]. Rather than maintaining a highly proliferative state, oxaliplatin-resistant cells may adopt a metabolically less active phenotype that promotes survival under chemotherapeutic stress. In CRC, metabolic reprogramming has been identified as a key determinant of both carcinogenesis and chemoresistance, enabling cancer cells to remodel their energy metabolism and maintain cellular homeostasis under adverse conditions [45].

In this study, parental HCT116 cells released a substantially higher number of hydrocarbons, particularly branched alkanes, compared with OXrHCT116 cells. The formation of alkanes has been widely linked to lipid peroxidation processes induced by oxidative stress, in which reactive oxygen species (ROS) attack PUFAs, resulting in the release of volatile degradation products such as ethane and n-pentane [46,47].

The elevated emission of these compounds in parental HCT116 cells may be consistent with increased oxidative stress and enhanced membrane turnover associated with rapid proliferation. In contrast, the reduced alkane release observed in OXrHCT116 cells suggests improved redox homeostasis and lower levels of lipid peroxidation. Such adaptations are consistent with known mechanisms of chemoresistance, in which enhanced antioxidant capacity and suppression of ROS-mediated cell damage contribute to survival under cytotoxic stress [48,49].

Additional differences were observed in the metabolism of aldehydes and alcohols. Parental HCT116 cells exhibited higher uptake of compounds such as 2-methylbutanal and 3-methylbutanal, which are known substrates of ALDHs and ADHs [50]. In contrast, oxaliplatin-resistant cells exhibited markedly reduced consumption of these metabolites, suggesting diminished enzymatic activity or metabolic flux through these pathways. This observation may reflect alterations in detoxification capacity and intracellular redox balance accompanying chemoresistance.

The emission of aromatic compounds was markedly reduced in OXrHCT116 cells. Although the biochemical origin of these VOCs remains poorly understood, reduced production of these compounds has been reported in several cancer cell lines, including lung and gastric cancers [15,40,51]. The diminished release of aromatic compounds in oxaliplatin-resistant cells may reflect altered enzymatic activity, potentially involving cytochrome P450 isoforms.

Interestingly, exposure to oxaliplatin induced alterations in the VOC profile of parental HCT116 cells, whereas the volatilome of OXrHCT116 cells remained largely unchanged. This finding suggests that OXrHCT116 cells possess a more stable metabolic phenotype that is less responsive to acute chemotherapeutic stress.

Several limitations of the present study should be acknowledged. First, the biological interpretation of the observed volatilomic alterations is based on indirect evidence. While VOCs represent downstream products of cellular metabolism and may provide valuable insights into underlying biochemical processes, intracellular metabolic pathways, oxidative stress, ROS production, lipid peroxidation, mitochondrial function, and established mechanisms of oxaliplatin resistance were not directly assessed. Furthermore, the biological origin of several VOCs remains uncertain, and alternative unknown metabolic pathways may contribute to their formation or consumption. The present findings were obtained under controlled in vitro conditions. VOC signatures in vivo reflect a complex interplay between tumor biology, host metabolism, microbiota, diet, medication use, and environmental exposures [52,53]. Therefore, the observed volatilomic differences, including the reduced VOC production in resistant cells, cannot be directly extrapolated to patient-derived samples. Consequently, the proposed mechanistic and clinical implications should be regarded as exploratory and hypothesis-generating. Future studies integrating volatilomics with intracellular metabolomics, transcriptomic analyses, ROS measurements, lipid peroxidation assays, assessments of mitochondrial function, functional metabolic analyses and clinical validation cohorts are required to clarify the biological and translational significance of the identified VOC signatures. Finally, the results of the Wilcoxon signed-rank test were not corrected for multiple comparisons. This resulted from the limited number of replicates possible to perform within the study.

Importantly, these findings underscore the potential clinical relevance of VOC analysis. As VOCs reflect real-time cellular metabolic activity, they represent attractive candidates for non-invasive biomarkers to monitor tumor behavior and therapeutic response. In the context of oxaliplatin resistance, the reduced VOC diversity and distinct emission patterns observed in OXrHCT116 cells suggest that volatilomic signatures may enable discrimination between therapy-sensitive and oxaliplatin-resistant phenotypes. Given that cancer-derived VOCs have already been detected in patient-derived samples such as breath, urine, and blood [13], VOC profiling may represent a promising complementary approach for the early detection of treatment resistance and for patient stratification in CRC. This concept is supported by recent advances in CRC volatilomics, including an AI-enhanced breath analysis platform that demonstrated the potential of VOC-based signatures for early-stage CRC detection [54]. Furthermore, recent studies have highlighted the importance of metabolic adaptations in oxaliplatin-resistant CRC including alterations in purine metabolism [55]. Additional investigations have identified profound transcriptional and metabolic changes accompanying the acquisition of oxaliplatin resistance, further supporting the concept that treatment resistance is closely linked to cellular metabolic plasticity [56]. Collectively, these findings support further investigation of volatilomic profiling as a complementary tool for the characterization and potentially the clinical monitoring of oxaliplatin-resistant colorectal cancer.

## 4. Materials and Methods

### 4.1. Chemicals and Standards

All calibration standards and reference mixtures were generated from high-purity liquid chemicals. As the detailed protocol for standard preparation has been described previously [57,58], only a brief outline is provided here. Reference compounds with declared purities ranging from 95 to 99.9% were purchased from Merck (Vienna, Austria) and Fluka (Buchs, Switzerland).

Reference standards were prepared in a two-step procedure. First, primary standards were produced by injecting and evaporating several microlitres of liquid compounds into pre-evacuated and heated 1 L glass bulbs (Supelco, Toronto, ON, Canada). Second, secondary standards were generated by transferring appropriate volumes of the primary standard into Tedlar bags (SKC Inc., Eighty Four, PA, USA) ranging in volume from 3 to 25 L, pre-filled with purified and humidified air (relative humidity 90–100% at 34 °C). Final calibration standards covering VOC volume fractions ranging from 0.06 to 70 parts per billion by volume (ppbv) were used for calibration and retention time determination. Calibration curves were constructed using 5 or 6 independent concentration levels.

### 4.2. Cell Lines

The human colon carcinoma cell line HCT116 was kindly provided by Prof. Brigitte Marian (Center of Cancer Research, Medical University of Vienna, Vienna, Austria) and authenticated by Microsynth AG (Balgach, Switzerland) prior to use. Routine testing for mycoplasma contamination was carried out (MycoSPY^®^, Biontex, Munich, Germany).

Oxaliplatin-resistant HCT116 cells (OXrHCT116) were generated in-house by continuous exposure to increasing concentrations of oxaliplatin (Merck, Darmstadt, Germany, product code: O9512-5MG), starting at 1 µM and gradually escalating to a final concentration of 2 µM over a period of approximately 2–3 months. No additional STR profiling of the resistant derivative was performed.

All cell lines were cultured under standard conditions at 37 °C in a humidified atmosphere containing 5% CO_2_ using Dulbecco’s Modified Eagle Medium (DMEM)/Ham’s F12 (1:1) medium (Thermo Fisher Scientific, Waltham, MA, USA) supplemented with 10% fetal calf serum (FCS; Thermo Fisher Scientific). To maintain the oxaliplatin-resistant phenotype, OXrHCT116 cells were cultured in medium supplemented with 2 µM oxaliplatin.

### 4.3. Cell Viability

Cell viability and dose–response were assessed using the 3-(4,5-dimethylthiazol-2-yl)-2,5-diphenyltetrazolium bromide (MTT)-based EZ4U cell proliferation and cytotoxicity assay (Biomedica, Vienna, Austria) according to the manufacturer’s instructions. Briefly, 1000 cells per well were seeded into 96-well plates and allowed to adhere overnight under standard culture conditions. The following day, the culture medium was replaced with fresh medium containing either no oxaliplatin or increasing concentrations of oxaliplatin (0.1, 1, 5, 10, 50, and 100 µM). Each concentration was tested in 6 replicates. After 72 h of treatment, EZ4U reagent was added to each well, and cells were incubated for an additional 4 h at 37 °C. Absorbance was subsequently measured using a BioTek 800 TS microplate reader (Agilent, Santa Clara, CA, USA) at 450 nm with a reference wavelength of 620 nm, according to the manufacturer’s protocol. Cell viability was expressed as a percentage relative to untreated control cells. Data represents pooled results from at least three independent viability assays.

### 4.4. Colony Formation

Single cells were seeded at low density (100 or 200 cells per well) into 6-well plates in cell culture medium. After 24 h of incubation, the medium was replaced with fresh growth medium to remove non-adherent cells. Cells were then cultured for an additional 7 days to allow colony formation. Colonies were washed with phosphate-buffered saline (PBS) and fixed with ice-cold methanol for at least 20 min. Following fixation, cells were washed again with PBS, stained with 0.01% crystal violet solution, and counted. Data represents pooled results from at least 3 independent colony formation assays. In this study, colony formation assays were performed in the absence of oxaliplatin.

### 4.5. Cell Lines Cultivation for VOC Measurements

For volatilomic analyses, 4 × 10^6^ HCT116 cells and 7 × 10^6^ OXrHCT116 cells were seeded into glass flasks (Ruprechter, Tyrol, Austria) and cultured for 72 h prior to VOC sampling. The glass flasks had dimensions of 21 × 5.5 × 11.5 cm^3^, corresponding to a nominal volume of 1 L and a bottom surface area of approximately 240 cm^2^. Flasks were sealed with an in-house manufactured cap that has been described in detail elsewhere [58]. The cap was constructed from inert materials (polytetrafluoroethylene (Teflon) and polyether ether ketone (PEEK)) exhibiting minimal VOC emissions and was equipped with a septum and a PEEK tube to allow the introduction of purified air and needle trap devices. During the cell cultivation period, the cap was kept slightly open to permit gas exchange. Twenty-four hours prior to VOC sampling, the flasks were hermetically sealed to allow accumulation of VOCs in the HS.

In total, 9 independent experiments were performed at different times. Within each experiment, the following were investigated: untreated HCT116 cells, untreated OXrHCT116 cells, HCT116 cells treated with 2 µM oxaliplatin, and OXrHCT116 cells treated with 2 µM oxaliplatin.

The total number of cells present in each flask at the start of measurement is summarized in Supplementary Materials Table S2. Cell numbers were comparable across individual replicates and varied around 50 × 10^6^. Viable cells were determined after trypsinization by manual counting using a Neubauer counting chamber. The applied experimental procedure did not adversely affect the cell viability.

In addition, control flasks containing culture medium with and without 2 µM oxaliplatin, but without cells, were processed under identical experimental conditions. Altogether, 9 sets of cultures (A-I) were prepared and used for comparative volatilomic analysis.

### 4.6. VOCs Extraction Protocol

Volatiles present in the HS above the culture medium alone or above cells cultured in the medium were pre-concentrated using HS-NTE. As the HS-NTE protocol applied in this study has been described in detail previously [57,58], only a brief summary is provided here. Two-bed, 23-gauge Silcosteel-treated stainless steel needle trap devices (NTDs) (PAS Technology, Germany), containing 2 cm of Carbopack X and 1 cm of Carboxen 1000 (both 60/80 mesh), were used for VOC collection. Prior to extraction, NTDs were pre-conditioned at 290 °C for 10 min under a flow of high purity nitrogen 6.0 (99.9999%). Needle trap extraction (NTE) was performed by introducing an NTD through a septum into the cultivation bottle and collecting 80 mL of the HS gas at a constant flow rate of 3 mL min^−1^. Following extraction, the NTD was inserted into the inlet of the gas chromatograph, where trapped volatiles were thermally desorbed at 290 °C in splitless mode. For each experimental replicate, a blank sample containing nitrogen was analyzed using the same protocol to identify potential contaminants originating from the sampling system. When applicable, background concentrations detected in the blank samples were subtracted from the corresponding HS sample values.

### 4.7. GC-MS Analysis

All analyses were performed using an Agilent 8890/7079B GC-MS (Agilent). The GC injector was equipped with an inert solid-phase microextraction (SPME) liner (internal diameter 0.75 mm; Supelco, Canada) and operated in splitless mode for 0.75 min, followed by the split mode at a ratio of 1:50. Chromatographic separation was achieved using a Rxi-624Sil MS column (30 m × 0.32 mm, layer thickness 1.8 μm; Restek, Centre County, PA, USA) operated in a constant helium flow of 1.4 mL min^−1^. The column temperature program was as follows: 37 °C for 12 min, followed by 5 °C min^−1^ to 150 °C, then 10 °C min^−1^ to 290 °C, with a final hold at 280 °C for 8 min. Untargeted VOC analysis was performed using the SCAN mode over an *m*/*z* range of 20–250. Peak integration was based on extracted *m*/*z* chromatograms, enabling effective separation of most of the analytes. The quadrupole analyzer, ion source, and transfer line temperatures were maintained at 150 °C, 230 °C, and 280 °C, respectively.

VOC identification relied on a two-step process. First, the mass spectrum of an unknown analyte was checked against the national institute of standards and technology (NIST) mass spectral library database. Second, the NIST identification was confirmed by matching the retention time with that obtained from reference standards analyzed under identical chromatographic conditions. However, for a subset of compounds, authentic reference standards were not available and confirmation by retention time was therefore not possible. Consequently, the identification of these compounds relied solely on mass spectral matching against the NIST library and should therefore be regarded as putative.

### 4.8. Validation Measures

An effort was made to quantify VOCs in all analyzed HSs. However, this was not possible for a subset of the compounds. This limitation was due either to the unavailability of pure reference substances or to difficulties in generating reliable reference volatile mixtures. For these VOCs, a linear detector response across the investigated concentration ranges was assumed, allowing relative comparisons based on peak areas alone. Thus, for these compounds all statistical tests were performed using their normalized peak areas rather than absolute concentrations.

Validation parameters of the HS-NTE-GC-MS method are presented in Supplementary Materials Table S3. Limits of detection (LOD) were calculated from the standard deviation of 5 blank measurements using the approach described by Huber [59]. The limit of quantification (LOQ) was defined as 3 × LOD and corresponds to the lower limit of the linear ranges. LODs ranged from 0.02 to 0.22 ppbv. Method precision, expressed as the relative standard deviation (RSD), ranged from 7 to 13%, which was considered acceptable for the purposes of this study. Linearity of the instrument response was evaluated by residual analysis. Specifically, residuals were tested for normality using the Anderson-Darling test, and a *p*-value < 0.05 was defined as the threshold for rejecting normality. Instrument response was linear over the investigated VOC concentration ranges, with coefficients of determination ranging between 0.979 and 0.998.

### 4.9. Statistical Analysis

Cell viability and colony formation data are presented as mean ± standard deviation (SD). Data normality was assessed using the Kolmogorov–Smirnov and Shapiro–Wilk tests. Depending on the distribution of the data, statistical differences between groups were evaluated using an unpaired two-tailed Student’s *t*-test with Welch’s correction for parametric data, or the Mann-Whitney U test for non-parametric data. Effect sizes for comparisons between HCT116 and OXrHCT116 cells were quantified using Cohen’s d, calculated as the difference between group means divided by the pooled standard deviation. All analyses were performed using Excel (version 2606) or GraphPad Prism (version 10.2.2; GraphPad Software, San Diego, CA, USA).

Dose response curves were fitted using nonlinear regression based on a four-parameter logistic model. Differences between dose–response curves were assessed using an extra sum-of-squares F-test. Half-maximal inhibitory concentration (IC_50_) values were calculated using GraphPad Prism (version 10.2.2).

Volatilomic analyses were performed by comparing the chemical composition of the HS above cell cultures with that of the medium alone. Differences were assessed using a Wilcoxon signed-rank test, with *p* < 0.05 considered to be statistically significant. To identify produced (released) VOCs, the respective net signal intensities (i.e., the VOC signal in the cell culture minus the VOC signal in the corresponding culture medium) were normalized to the cell number at the time of sampling. In contrast, metabolized VOCs were identified using unnormalized net signal intensities because, in a number of cultures, the VOCs of interest were depleted to concentrations below the limit of detection (LOD) of the applied method, rendering per-cell normalization biologically inappropriate. Effect size (*r*) was calculated for the Wilcoxon signed-rank test.

## 5. Conclusions

Taken together, the volatilomic signature of OXrHCT116 cells is characterized by reduced VOC diversity, diminished emission of lipid peroxidation-associated compounds, and decreased substrate turnover. These features align closely with the observed reduction in proliferation and clonogenicity, indicating a shift toward a metabolically less active yet more stress-resilient cellular phenotype.

This study highlights the potential of VOC analysis as a non-invasive approach to capture metabolic adaptations associated with chemoresistance. Although the precise biochemical origins of several detected compounds remain to be fully elucidated, the consistent differences observed between oxaliplatin-sensitive and oxaliplatin-resistant cells underscore the value of volatilomic profiling for advancing our understanding of tumor metabolism and therapy response. Future studies integrating VOC analysis with intracellular metabolomics, transcriptomic profiling, ROS measurements, and assessments of mitochondrial function will be essential to validate the proposed biological mechanisms and further clarify the links between volatilomic signatures and resistance phenotypes. In a clinical context, VOC-based profiling may provide a minimally invasive strategy for monitoring treatment response and could potentially enable early identification of oxaliplatin resistance in CRC patients, thereby supporting improved patient stratification and personalized therapeutic decision-making.

## Figures and Tables

**Table 1:** Consumption of volatile organic compounds (VOCs) by HCT116 and OXrHCT116 cells relative to culture medium controls and their tentative metabolic pathways of consumption. The proposed pathways are based on published literature regarding human VOC metabolism and are provided for biological interpretation only. The identification of VOCs in brackets was not confirmed by the retention time library. Compounds shown in italics were not quantified; statistical analyses for these compounds were therefore performed using peak areas. CAS: Chemical Abstracts Service registry number; ADHs: alcohol dehydrogenases; ALDHs: aldehyde dehydrogenases; AOXs: aldehyde oxidases; XOs: xanthine oxidases; n.s.: not significant.

VOC	CAS	HCT116	OXrHCT116	HCT116 + Oxaliplatin	OXrHCT116 + Oxaliplatin	Proposed Metabolic Pathways Based on Published Literature
		*p*-Value (Effect Size r)	*p*-Value (Effect Size r)	*p*-Value (Effect Size r)	*p*-Value (Effect Size r)	Tentative Metabolic Product(s) (Enzymes/Processes)
Butanal, 3-methyl-	590-86-3	9.2 × 10^−3^ (0.91)	9.2 × 10^−3^ (0.91)	n.s.	9.2 × 10^−3^ (0.91)	I. 3-methylbutanol (ADHs) II. 3-methylbutanoic acid (ALDHs/AOXs/XOs)
Butanal, 2-methyl-	96-17-3	3.6 × 10^−2^ (0.94)	3.6 × 10^−2^ (0.94)	n.s.	n.s.	I. 2-methylbutanol (ADHs) II. 2-methylbutanoic acid (ALDHs/AOXs/XOs)
*(1,3-Dioxan-5-ol)*	*4740-78-7*	*2.3 × 10^−2^* *(0.94)*	*n.s.*	*2.3 × 10^−2^* *(0.94)*	*n.s.*	
*1-Hexene, 4,5-dimethyl-*	*16106-59-5*	*3.6 × 10^−2^* *(0.94)*	*n.s.*	*n.s.*	*n.s.*	*Oxidation (cytochrome P450 isoforms)*
*(1-Octene, 3,4-dimethyl-)*	*56728-11-1*	*2.4 × 10^−2^* *(0.93)*	*n.s.*	*1.4 × 10^−2^* *(0.91)*	*n.s.*	*Oxidation (cytochrome P450 isoforms)*
*(2,4-Hexadien-1-ol)*	*111-28-4*	*n.s.*	*2.3 × 10^−2^* *(0.92)*	*n.s.*	*9.2 × 10^−3^* *(0.92)*	*2,4-Hexadienal (ADHs/cytochrome P450 + ALDHs/AOXs/XOs)*
*(1-Decene, 3,4-dimethyl-)*	*50871-03-9*	*2.3 × 10^−2^* *(0.92)*	*n.s.*	*n.s.*	*n.s.*	*Oxidation (cytochrome P450 isoforms)*
*(1-Octanol, 2-butyl-)*	*3913-02-8*	*3.3 × 10^−2^* *(0.75)*	*n.s.*	*n.s.*	*n.s.*	*2-butyloctanal (ADHs/cytochrome P450 + ALDHs/AOXs/XOs)*
*(1-Octyn-3-ol, 4-ethyl-)*	*5877-42-9*	*3.6 × 10^−2^* *(0.94)*	*n.s.*	*3.5 × 10^−2^* *(0.86)*	*3.6 × 10^−2^* *(0.94)*	*Unknown (ADHs/cytochrome P450)*
*(Decane, 2,3,5,8-tetramethyl-)*	*192823-15-7*	*n.s.*	*n.s.*	*1.4 × 10^−2^* *(0.86)*	*n.s.*	
*(5-Tridecene, (Z)-)*	*25524-42-9*	*n.s.*	*n.s.*	*n.s.*	*3.6 × 10^−2^* *(0.94)*	*Oxidation (cytochrome P450 isoforms)*

**Table 2:** Emission of volatile organic compounds (VOCs) by HCT116 and OXrHCT116 cells normalized to cell count at the time of sampling, relative to culture medium controls, and their tentative metabolic pathways of production. The proposed pathways are based on published literature regarding human VOC metabolism and are provided for biological interpretation only. The identification of VOCs in brackets was not confirmed by the retention time library. Compounds shown in italics were not quantified; statistical analyses for these compounds were therefore performed using peak areas. CAS: Chemical Abstracts Service registry number; ADHs: alcohol dehydrogenases; PUFA: polyunsaturated fatty acids; n.s.: not significant.

VOC	CAS	HCT116	OXrHCT116	HCT116 + Oxaliplatin	OXrHCT116 + Oxaliplatin	Proposed Metabolic Pathways Based on Published Literature
		*p*-Value (Effect Size r)	*p*-Value (Effect Size r)	*p*-Value (Effect Size r)	*p*-Value (Effect Size r)	
n-Pentane	109-66-0	9.8 × 10^−3^ (0.75)	n.s.	2 × 10^−3^ (0.87)	1.4 × 10^−2^ (0.71)	Peroxidation of PUFA (oxidative stress)
*Acetone*	*67-64-1*	*2.7 × 10^−2^* *(0.63)*	*2 × 10^−3^* *(0.87)*	*1.9 × 10^−2^* *(0.67)*	*5.9 × 10^−3^* *(0.79)*	*I. 2-Propanol (ADHs/cytochrome P450 CYP2E1)* *II. Acetoacetate (spontaneous decarboxylation)*
2-Propanol, 2-methyl-	75-65-0	7.8 × 10^−3^ (0.86)	2 × 10^−3^ (0.87)	1.6 × 10^−2^ (0.8)	1.9 × 10^−3^ (0.86)	I. 2-Methoxy-2-methylpropane/2-ethoxy-2-methyl-propane (monooxygenases, e.g., cytochrome P450 2A6) II. 2-Methylpropane (cytochrome P450 isoforms (CYP1A2, CYP2B6, and CYP2E1))
*1-Propanol*	*71-23-8*	*3.7 × 10^−2^* *(0.59)*	*n.s.*	*n.s.*	*n.s.*	I. Propanal (ADHs)
Propane, 2-ethoxy-2-methyl-	637-92-3	7.8 × 10^−3^ (0.82)	1.9 × 10^−3^ (0.87)	n.s.	n.s.	
2-Butanone	78-93-3	1.4 × 10^−2^ (0.71)	3.9 × 10^−3^ (0.83)	n.s.	n.s.	I. 2-butanol (ADHs/cytochrome P450 CYP2E1) II. Fatty acids (β-oxidation)
*Benzene*	*71-43-2*	*1.9 × 10^−3^* *(0.87)*	*n.s.*	*1.9 × 10^−3^* *(0.87)*	*3.7 × 10^−3^* *(0.59)*	
Ethyl acetate	141-78-6	1.9 × 10^−3^ (0.87)	1.9 × 10^−3^ (0.87)	3.9 × 10^−3^ (0.87)	1.9 × 10^−3^ (0.87)	Ethanol + acetic acid (esterification)
*Hexane, 3-methyl-*	*589-34-4*	*n.s.*	*n.s.*	*7.8 × 10^−3^* (0.87)	*1.6 × 10^−2^* (0.86)	*Peroxidation of PUFA (oxidative stress)*
*(Ethane, 1,2-dichloro-)*	*107-06-2*	*9.8 × 10^−3^* *(0.75)*	*n.s.*	*n.s.*	*n.s.*	
1-Propanol, 2-methyl-	78-83-1	1.9 × 10^−3^ (0.87)	3.9 × 10^−3^ (0.87)	3.9 × 10^−3^ (0.87)	3.9 × 10^−3^ (0.87)	I. 2-methyl-propanal (ADHs)
2-Butanol, 2-methyl-	75-85-4	1.9 × 10^−3^ (0.87)	3.9 × 10^−3^ (0.87)	7.8 × 10^−3^ (0.87)	1.9 × 10^−3^ (0.87)	I. Tert-amyl methyl ether (monooxygenase, e.g., cytochrome P450) II. 2-Methylbutane (cytochrome P450 isoforms (CYP1A2, CYP2B6, and CYP2E1))
n-Heptane	142-82-5	4.9 × 10^−2^ (0.55)	4.9 × 10^−2^ (0.55)	*n.s.*	*n.s.*	Peroxidation of PUFA (oxidative stress)
2-Pentanone	107-87-9	1.9 × 10^−3^ (0.87)	1.9 × 10^−3^ (0.87)	1.9 × 10^−3^ (0.87)	1.9 × 10^−3^ (0.87)	I. 2-pentanol (ADHs/cytochrome P450 CYPE1) II. Fatty acids: hexanoic acid (β-oxidation)
3-Pentanone	96-22-0	3.9 × 10^−3^ (0.87)	n.s.	n.s.	n.s.	I. 3-pentanol (ADHs/cytochrome P450 CYP2E1) II. 2-methyl-3-ketovaleric acid (propionyl-CoA/methylmalonyl-CoA)
Ethyl propanoate	105-37-3	3.1 × 10^−2^ (0.84)	1.6 × 10^−2^ (0.86)	1.6 × 10^−2^ (0.86)	1.6 × 10^−2^ (0.86)	Ethanol + propanoic acid (esterification)
*(Pentane, 2,3,4-trimethyl-)*	*565-75-3*	*7.8 × 10^−3^* *(0.86)*	*n.s.*	*7.8 × 10^−3^* *(0.86)*	*n.s.*	*Peroxidation of PUFA (oxidative stress)*
*Heptane, 3-methyl-*	*589-81-1*	*1.9 × 10^−3^* *(0.86)*	*3.1 × 10^−2^* *(0.84)*	*3.9 × 10^−3^* *(0.86)*	*n.s.*	*Peroxidation of PUFA (oxidative stress)*
n-Octane	111-65-9	1.9 × 10^−3^ (0.86)	9.7 × 10^−3^ (0.75)	1.9 × 10^−3^ (0.86)	n.s.	Peroxidation of PUFA (oxidative stress)
*Ethane, 1,1-diethoxy-*	*105-57-7*	*1.9 × 10^−3^* *(0.86)*	*3.1 × 10^−2^* *(0.85)*	*3.9 × 10^−3^* *(0.86)*	*n.s.*	
*(Pentane, 2,3,3-trimethyl-)*	*560-21-4*	*7.8 × 10^−3^* *(0.86)*	*3.1 × 10^−2^* *(0.86)*	*1.6 × 10^−2^* *(0.86)*	*n.s.*	*Peroxidation of PUFA (oxidative stress)*
2-Pentanone, 4-methyl-	108-10-1	7.8 × 10^−3^ (0.82)	7.8 × 10^−3^ (0.82)	7.8 × 10^−3^ (0.82)	n.s.	4-Methyl-2-pentanol (ADHs/cytochrome P450 CYP2E1)
*Toluene*	*108-88-3*	*3.9 × 10^−3^* *(0.82)*	*n.s.*	*n.s.*	*n.s.*	
*(2,4-Dimethyl-1-heptene)*	*19549-87-2*	*3.7 × 10^−2^* *(0.59)*	*n.s.*	*1.9 × 10^−2^* *(0.69)*	*n.s.*	
*Heptane, 2,3-dimethyl*-	*3074-71-3*	*3.9 × 10^−3^* *(0.82)*	*n.s.*	*n.s.*	*n.s.*	*Peroxidation of PUFA (oxidative stress)*
Ethyl 2-methylbutyrate	7452-79-1	n.s.	n.s.	1.6 × 10^−2^ (0.86)	1.6 × 10^−2^ (0.86)	Ethanol + 2-methylbutanoic acid (esterification)
*Ethylbenzene*	*100-41-4*	*1.9 × 10^−3^* (0.86)	*n.s.*	*3.9 × 10^−3^* (0.86)	*n.s.*	
*Benzene, 1,3-dimethyl-*	*108-38-3*	*n.s.*	*7.8 × 10^−3^* *(0.86)*	*n.s.*	*n.s.*	
*o-Xylene*	*95-47-6*	*1.6 × 10^−2^* *(0.86)*	*n.s.*	*n.s.*	*n.s.*	
*n-Nonane*	*111-84-2*	*3.9 × 10^−3^* *(0.86)*	*3.9 × 10^−3^* *(0.86)*	*7.8 × 10^−3^* *(0.86)*	*7.8 × 10^−3^* *(0.86)*	*Peroxidation of PUFA (oxidative stress)*
*Styrene*	*100-42-5*	*3.9 × 10^−3^* *(0.86)*	*7.8 × 10^−3^* *(0.81)*	*3.9 × 10^−3^* *(0.86)*	*n.s.*	
2-Heptanone	110-43-0	7.8 × 10^−3^ (0.86)	7.8 × 10^−3^ (0.86)	7.8 × 10^−3^ (0.86)	3.1 × 10^−2^ (0.86)	I. 2-heptanol (ADHs/cytochrome P450 CYP2E1) II. Fatty acids: 2-ethylhexanoic acid (β-oxidation)
*3-Carene*	*13466-78-9*	*n.s.*	*n.s.*	*1.6 × 10^−2^* *(0.86)*	*n.s.*	
*Cyclohexanone*	*108-94-1*	*n.s.*	*n.s.*	*1.9 × 10^−3^* *(0.86)*	*n.s.*	*Cyclohexanol (ADHs)*
*(Octane, 4-ethyl-)*	*15869-86-0*	*1.9 × 10^−3^* *(0.86)*	*n.s.*	*n.s.*	*n.s.*	*Peroxidation of PUFA (oxidative stress)*
*(Octane, 4,5-diethyl-)*	*1636-41-5*	*1.9 × 10^−3^* *(0.86)*	*n.s.*	*n.s.*	*n.s.*	*Peroxidation of PUFA (oxidative stress)*
*Nonane, 3-methyl-*	*5911-04-6*	*3.9 × 10^−3^* *(0.86)*	*n.s.*	*n.s.*	*n.s.*	*Peroxidation of PUFA (oxidative stress)*
*Heptane, 2,2,4,6,6-pentamethyl-*	*13475-82-6*	*4.9 × 10^−2^* *(0.55)*	*n.s.*	*5.9 × 10^−3^* *(0.79)*	*1.9 × 10^−2^* *(0.71)*	*Peroxidation of PUFA (oxidative stress)*
*(Nonane, 2,6-dimethyl-)*	*17302-28-2*	*1.6 × 10^−2^* *(0.79)*	*n.s.*	*n.s.*	*n.s.*	*Peroxidation of PUFA (oxidative stress)*
*Benzene, 1,2,3-trimethyl-*	*526-73-8*	*1.9 × 10^−2^* *(0.72)*	*n.s.*	*n.s.*	*n.s.*	
1-Hexanol, 2-ethyl-	104-76-7	4.9 × 10^−2^ (0.55)	3.9 × 10^−3^ (0.83)	n.s.	2.7 × 10^−2^ (0.63)	I. Di(2-ethylhexyl)phthalate (CEase, Ces1e) II. 2-ethylhexanal (ADHs)
*n-Dodecane*	*112-40-3*	*n.s.*	*n.s.*	*2.7 × 10^−2^* *(0.63)*	*n.s.*	*Peroxidation of PUFA (oxidative stress)*
*n-Tetradecane*	*629-59-4*	*n.s.*	*n.s.*	*4.8 × 10^−2^* *(0.55)*	*2.7 × 10^−2^* *(0.63)*	Peroxidation of PUFA (oxidative stress)
*(3-Chloropropionic acid, heptadecyl ester)*	*1000283-05-1*	*n.s.*	*1.6 × 10^−2^* *(0.86)*	*n.s.*	*n.s.*	

## Data Availability

The original contributions presented in this study are included in the article/Supplementary Materials. Further inquiries can be directed to the corresponding authors.

## Supplementary Materials

The following supporting information can be downloaded at https://www.mdpi.com/article/10.3390/ijms27146240/s1.

## Abbreviations

The following abbreviations are used in this manuscript:

VOC	Volatile organic compound
OXrHCT116	Oxaliplatin-resistant HCT116
HS-NTE	Headspace needle trap extraction
GS-MS	Gas chromatography—Mass spectrometry
IC50	Half maximal inhibitory concentration
CRC	Colorectal cancer
FOLFOX	Folinic acid, Fluorouracil, Oxaliplatin
ppbv	Parts per billion by volume
FCS	Fetal calf serum
DMEM	Dulbecco’s modified eagle medium
MTT	3-(4,5-dimethylthiazol-2-yl)-2,5-diphenyltetrazolium bromide
PBS	Phosphate-buffered saline
PEEK	Polyether ether ketone
HS	Headspace
NTD	Needle trap devices
SPME	Solid-phase microextraction
NIST	National institute of standards and technology
LOD	Limits of detection
LOQ	Limits of quantification
SD	Standard deviation
ALDH	Aldehyde dehydrogenase
AOX	Aldehyde oxidase
XO	Xanthine oxidoreductase
ADH	Alcohol dehydrogenase
PUFA	Polyunsaturated fatty acid
DEHP	Di(2-ethylhexyl) phthalate
MEHP	Mono(2-ethylhexyl) phthalate
Cease	Cholesterol esterase
ROS	Reactive oxygen species
CAS	Chemical abstracts service
Rt	Retention times
RSD	Relative standard deviation
R^2^	Coefficients of determination
A-D	Anderson-Darling normality test
nd	Number of detections
nq	Number of quantified samples
n.s.	Not significant
