# Supplementary material for: Volatilomic Signatures of Parental and Oxaliplatin-Resistant HCT116 Colon Cancer Cell Lines
Source: Int J Mol Sci. 2026 Jul 13;27(14):6240. doi: 10.3390/ijms27146240 (PMC13410231; doi:10.3390/ijms27146240)
Supplement: Supplementary file 1 [file ijms-27-06240-s001.zip › ijms-4354250-supplementary.pdf]

Supplementary materials

Table S1. Detection and quantification incidences, concentration ranges, and median concentrations of calibrated volatile organic compounds (VOCs) in the headspace (HS) of culture media and cell cultures under basal conditions and after oxaliplatin exposure. Compounds are listed in order of increasing retention time. CAS: Chemical Abstracts Service registry number; nd: number of detections; nq: number of quantified samples.

	VOC	CAS	HCT116		OXrHCT116		HCT116 + oxaliplatin		OXrHCT116 + oxaliplatin		Medium		Medium + oxaliplatin	
			nd(nq)	Range (median) [ppb]	nd(nq)	Range (median) [ppb]	nd(nq)	Range (median) [ppb]	nd(nq)	Range (median) [ppb]	nd(nq)	Range (median) [ppb]	nd(nq)	Range (median) [ppb]
**Uptake**	Butanal, 3-methyl-	590-86-3	2(3)	2.06-2.38 (2.22)	1(1)	1.86	3(3)	1.94-6.05 (2.73)	0(0)	-	9(9)	1.93-9.23 (2.2)	9(9)	1.9-8.1 (2.2)
	Butanal, 2-methyl-	96-17-3	0(0)	-	4(4)	0.13-1.91 (0.19)	4(4)	0.13-2.1 (0.3)	4(2)	0.19-1.52 (0.86)	9(9)	2.56-18 (3.1)	4(4)	2.86-13.9 (3.42)
**Release**	2-Propanol, 2-methyl-	75-65-0	7(7)	0.25-3.56 (0.53)	9(9)	0.15-3.29 (0.49)	7(7)	0.25-294 (0.48)	9(9)	0.14-2.92 (0.5)	4(4)	0.22-2.53 (1.14)	3(3)	0.35-2.5 (2.09)
	Propane, 2-ethoxy-2-methyl-	637-92-3	8(8)	1.94-17.1 (2.96)	9(9)	1.92-9.07 (2.76)	8(8)	2.06-10.9 (3.33)	7(7)	2.08-4.65 (2.6)	3(3)	1.96-5.83 (2.15)	5(5)	1.97-7.17 (4.47)
	2-Butanone	78-93-3	9(9)	22.9-404 (73.5)	9(9)	18.6-414 (85.5)	8(8)	20.4-599 (58.9)	7(7)	17.3-383 (56.5)	9(9)	13.6-295 (64.8)	9(9)	11.1-977 (68.7)
	Ethyl acetate	141-78-6	9(9)	0.63-22.4 (1.44)	9(9)	0.54-27.4 (2.48)	8(8)	0.39-21.6 (1.37)	7(7)	0.49-124 (19.6)	9(8)	0.19-2.0 (0.47)	5(5)	0.16-1.35 (0.22)
	1-Propanol, 2-methyl-	78-83-1	9(9)	0.47-9.52 (1.04)	8(8)	0.35-5.47 (1.24)	8(8)	0.32-5.68 (0.88)	8(8)	0.21-5.61 (0.96)	4(4)	0.47-3.43 (2.38)	2(2)	2.69-3.53 (3.11)
	2-Butanol, 2-methyl-	75-85-4	9(7)	0.31-3.45 (0.58)	8(7)	0.32-1.98 (0.53)	7(6)	0.41-2.51 (0.48)	9(7)	0.3-1.63 (0.44)	0(0)	-	0(0)	-
	2-Pentanone	107-87-9	9(9)	0.36-22 (0.94)	9(9)	0.26-19.5 (1.01)	9(9)	0.33-17.3 (0.79)	9(9)	0.26-16.1 (0.92)	7(4)	0.34-3.75 (0.76)	8(4)	0.36-2.58 (0.8)
	3-Pentanone	96-22-0	8(8)	0.13-2.72 (0.19)	4(4)	0.13-1.91 (0.2)	4(4)	0.13-2.1 (0.3)	4(4)	0.12-1.52 (0.86)	2(2)	0.1-1.24 (0.67)	4(2)	0.1-0.14 (0.12)

	Ethyl propanoate	105-37-3	5(5)	0.36-22.1 (0.94)	6(6)	0.21-7.9 (0.48)	6(6)	0.24-6.16 (1.52)	6(6)	0.24-7.0 (1.94)	0(0)	-	0(0)	-
	n-Octane	111-65-9	9(9)	2.55-33.8 (3.97)	9(9)	2.49-18.2 (2.95)	9(9)	2.47-19.3 (3.59)	9(9)	2.36-8.44 (2.72)	6(6)	2.26-11.5 (3.23)	6(6)	2.38-9.0 (3.24)
	Ethyl 2-methylbutyrate	7452-79-1	3(1)	2.25	4(4)	0.07-1.9 (0.1)	6(4)	0.23-1.42 (0.47)	6(6)	0.06-1.45 (0.45)	0(0)	-	0(0)	-
	2-Heptanone	105-42-0	7(6)	0.15-4.22 (0.2)	7(7)	0.12-3.0 (0.16)	7(6)	0.14-3.23 (0.12)	5(4)	0.12-2.37 (0.22)	0(0)	-	0(0)	-
	1-Hexanol, 2-ethyl-	104-76-7	9(9)	2.4-186 (6.1)	8(8)	2.7-161.8 (4.8)	8(8)	0.24-6.16 (1.52)	8(8)	3.26-151 (4.34)	8(8)	1.66-91 (3.0)	6(6)	1.94-45.4 (2.67)

Table S2. Total number of cells [×10^6] in the cultivation flasks at the time of the measurement.

Cell line	Total number of cells [×10^6]									
	**A**	**B**	**C**	**D**	**E**	**F**	**G**	**H**	**I**	**Median**
HCT116	48.8	77.4	62.4	74.6	61.0	41.3	46.2	64.5	53.8	**61.0**
OXrHCT116	66.1	82.7	50.7	48.1	55.8	54.1	36.7	69.0	65.5	**55.8**
HCT116 + oxaliplatin	29.2	83.9	43.8	48.9	58.4	38.8	29.2	42.8	37.4	**42.8**
OXrHCT116 + oxaliplatin	42.4	78.7	51.3	33.1	45.2	53.5	40.4	59.8	71.0	**51.3**

Table S3. Retention times (Rt), quantifier ions, limits of detection (LOD), relative standard deviations (RSD), coefficients of determination (R^2), Anderson–Darling normality test p-values (A–D), and linear ranges of the analyzed volatile organic compounds (VOCs). Compounds are listed in order of increasing retention time. CAS: Chemical Abstracts Service registry number.

VOC	CAS	Rt [min]	Quantifier ion	LOD [ppb]	RSD [%]	R2	A-D p-value	Linear Range [ppb]
2-Propanol, 2-methyl-	75-65-0	4.17	59	0.04	9.0	0.995	0.25	0.13-20
Propane, 2-ethoxy-2-methyl-	637-92-3	6.13	59	0.05	13	0.990	0.27	0.16-19
2-Butanone	78-93-3	6.50	72	0.22	10	0.989	0.27	0.73-60
Ethyl acetate	141-78-6	6.71	43	0.04	7.0	0.983	0.14	0.12-13
1-Propanol, 2-methyl-	78-83-1	9.40	43	0.13	13	0.997	0.14	0.46-20
2-Butanol, 2-methyl-	75-85-4	9.80	59	0.09	13	0.994	0.27	0.3-19
Butanal, 3-methyl-	590-86-3	9.97	58	0.06	9.0	0.981	0.27	0.17-50
Butanal, 2-methyl-	96-17-3	10.57	57	0.02	9.0	0.990	0.28	0.12-30
2-Pentanone	107-87-9	13.30	43	0.08	12	0.995	0.13	0.26-28
3-Pentanone	96-22-0	14.03	57	0.03	13	0.990	0.15	0.09-20
Ethyl propanoate	105-37-3	14.20	57	0.04	11	0.979	0.34	0.12-13
2-Pentanone, 4-methyl-	108-10-1	17.30	43	0.07	12	0.987	0.07	0.2-17
1-Butanol, 3-methyl-	123-51-3	18.20	55	0.07	12	0.994	0.16	0.23-17
n-Octane	111-65-9	18.48	85	0.01	14	0.994	0.21	0.03-13

Ethyl 2-methylbutyrate	7452-79-1	22.54	102	0.03	11	0.982	0.36	0.09-21
2-Heptanone	105-42-0	25.17	58	0.04	13	0.988	0.11	0.12-11
1-Hexanol, 2-ethyl-	104-76-7	30.73	57	0.04	7.0	0.998	0.23	0.12-60
